# Al-Ce co-doped BaTiO_3_ nanofibers as a high-performance bifunctional electrochemical supercapacitor and water-splitting electrocatalyst

**DOI:** 10.1038/s41598-024-54561-4

**Published:** 2024-04-29

**Authors:** Fatemeh Zakeri, Abbas Javid, Yasin Orooji, Arezou Fazli, Amirreza Khataee, Alireza Khataee

**Affiliations:** 1 College of Materials Science and Engineering, Co-Innovation Center of Efficient Processing and Utilization of Forest Resources, Nanjing Forestry University, No. 159, Longpan Road, Nanjing, 210037 Jiangsu China; 2 Research Laboratory of Advanced Water and Wastewater Treatment Processes, Department of Applied Chemistry, Faculty of Chemistry, University of Tabriz, Tabriz, 51666-16471 Iran; 3 College of Geography and Environmental Sciences, Zhejiang Normal University, Jinhua, 321004 China; 4 Smart Materials, Istituto Italiano di Tecnologia, via Morego 30, 16163 Genoa, Italy; 5 Division of Applied Electrochemistry, Department of Chemical Engineering, KTH Royal Institute of Technology, 100 44 Stockholm, Sweden; 6 Department of Chemical Engineering, Istanbul Technical University, Istanbul, 34469 Turkey

**Keywords:** BaTiO_3_ nanofibers, Supercapacitor, Pseudo-capacitor, Piezo-electric potential, Oxygen evolution reaction, Hydrogen evolution reaction, Overall water splitting, Environmental sciences, Environmental chemistry, Environmental impact

## Abstract

Supercapacitors and water splitting cells have recently played a key role in offering green energy through converting renewable sources into electricity. Perovskite-type electrocatalysts such as BaTiO_3_, have been well-known for their ability to efficiently split water and serve as supercapacitors due to their high electrocatalytic activity. In this study, BaTiO_3_, Al-doped BaTiO_3_, Ce-doped BaTiO_3_, and Al-Ce co-doped BaTiO_3_ nanofibers were fabricated via a two-step hydrothermal method, which were then characterized and compared for their electrocatalytic performance. Based on the obtained results, Al-Ce co-doped BaTiO_3_ electrode exhibited a high capacitance of 224.18 Fg^−1^ at a scan rate of 10 mVs^−1^, high durability during over the 1000 CV cycles and 2000 charge–discharge cycles, proving effective energy storage properties. Additionally, the onset potentials for OER and HER processes were 11 and − 174 mV vs. RHE, respectively, demonstrating the high activity of the Al-Ce co-doped BaTiO_3_ electrode. Moreover, in overall water splitting, the amount of the overpotential was 0.820 mV at 10 mAcm^−2^, which confirmed the excellent efficiency of the electrode. Hence, the remarkable electrocatalytic performance of the Al-Ce co-doped BaTiO_3_ electrode make it a promising candidate for renewable energy technologies owing to its high conductivity and fast charge transfer.

## Introduction

The rapidly growing modern society and civilization caused increasing energy consumption and a global environmental crisis. Coal and oil consumption to supply the needed energy release a huge amount of carbonaceous gases into the environment, causing global concerns. Hence, scientists around the world have been focusing on developing energy conversion and storage apparatus and exploiting renewable energy systems rather than traditional fossil fuels^1,2^.

According to previous researches, ultracapacitors or supercapacitors as an electrochemical energy storage method are the most endurable and proper electrochemical energy storage and converters to electrical energy mechanism among the diverse energy storage methods. Because of their high energy and power density, stability and fast charge–discharge capability, they are widely applied in many portable electronic and microelectronic devices. Based on the charge storage processes, supercapacitors can be classified into two main groups: electric double-layer capacitive (EDLC) that exhibit non-faradaic electrochemistry and pseudocapacitive that indicate faradaic electrochemistry^2–4^. Moreover, pseudocapacitors possess a higher energy density compared to EDLCs, which has motivated scientists to study them^5^. The morphology and configuration of the active electrode materials play a crucial role in achieving the appropriate capacitance, power, and energy densities. On the other hand, the oxygen evolution reaction (OER) and hydrogen evolution reaction (HER) are the main reactions in renewable and clean energy conversion systems. These two half-reactions form the electro and photocatalytic water splitting profiting from abundant water sources^6^. Additionally, the charge transfer and separation, light absorption and hydrogen generation are seriously limited by the rate controlling of the photocatalytic water splitting processes due to its slow electron transport and easy holes and electrons recombination^7^. Because of these deficiencies, great attention has been focused on the electrocatalytic water splitting process. In order to increase the efficiency of the OER and HER reaction and overcome the high overpotential (η) in the overall water splitting, a cost-effective and highly active electrocatalyst are required^7^.

Recent researchers have used valuable and rare earth metal electrodes such as Ru, Ir, and Pt because of their excellent electrocatalytic efficiency. However, their high price, paucity, and instability hamper their practical applications^8^. Hence, it is necessary to fabricate a cost-effective electrocatalyst with high electrocatalytic performance and nonprecious metal to increase the electrocatalytic activity^9^. Over the past few decades, perovskite oxide materials (ABO_3_) have attracted rapidly increasing attention due to their excellent properties, such as high stability, oxygen vacancies, high electrocatalytic activity, affordable raw materials, and environmental friendliness^10^. Further, perovskite oxide materials exhibit large specific pseudocapacitance due to multi-electron transfer during the redox reactions, as well as high-power density and better stability than the organic electrochemical double-layer capacitor electrodes^11^. In this regard, BaTiO_3_ has been recognized as a perovskite oxide material and employed as an electrode in electrochemical energy storage and conversion devices due to its inherent oxygen vacancies and high conductivity^12^.

Elemental doping is a convenient and effective method to form oxygen vacancies, modify the crystal structure of materials, thereby leading to alterations in their physico-chemical and charge movement properties^13^. Hence, extensive research has focused on A- and B-site doping modification due to their experimental feasibility and there potential to influence various aspects of the material. Accordingly, these modifications can impact the structure, the gate size, the oxygen vacancy concentration, and the metal–oxygen average binding energy, ultimately leading to an improved performance of the perovskites^14^. However, the effects cannot be strictly categorized as A- or B-site alterations; rather, the predominant role is played by the involved defect reaction^15^.

It is well established that the electrical properties of BaTiO_3_ materials can be modified through doping or co-doping various elements at A-site Ba^2+^ or B-site Ti^4+^. In addition, doped BaTiO_3_ effectively promote the formation of active redox sites, generation of oxygen vacancies, the response rates of electrode–electrolyte diffusion, and performance metrics of energy and power density^16^. This, in turn, contributes to increase pseudocapacitance, facilitate ion transfer, and enhance cycling durability^17^. In a recent study by Tanwar et al., Eu-doped BaTiO_3_ perovskite exhibited enhanced OER electrocatalysis, demonstrating improved durability and performance compared to bare BaTiO_3_. This enhancement is attributed to increased active sites and enhanced conductivity from the Eu ion. The Tafel slope measured 138.34 mV dec^−1^, with a low overpotential of 532 mV vs. SCE at a current density of 10 mA cm^−2^ in a 1.0 M KOH solution. In another study, Artrith et al. synthesized Fe- and Ni-modified BaTiO3 for electrocatalytic water splitting, revealing a low bulk resistance of about 7.5 MΩ, in contrast to the pristine BaTiO_3_ resistance of approximately 0.13 GΩ.

Density functional theory reports suggest that the redox kinetics of an electrode material can be promoted without compromising the catalytic activity, through alloying it with a small amount of a rare earth metal. Therefore, rare earth elements are not only beneficial for phase stability, they also offer superior and stable electrochemical properties by changing the lattice parameters^17,18^. Cerium has attained a significant interest among different rare earth metals, particularly due to its significant impact when simultaneously occupancy at Ba and Ti sites^15^. The high redox active Ce ions, stemming from the various 4f states of electronic structures during redox process, enrich spontaneous polarization, raise the specific capacitance characteristics, and improve the efficiency in both OER and HER^13,16,19^. On the other hand, Ce element doping creates the oxygen vacancies resulting in an improved conductivity and consequently promote the electrochemical behaviors of the electrocatalyst^19,20^. For instance, P. Senthilkumar and coworkers^19^, describe that the Ce doped BaTiO_3_ Nanoassemblies photoanode displays the high water splitting performance and decreases the onset potential from − 0.726 to − 0.504 V compare to undoped BaTiO_3_. In addition, Liu et al.^21^ evaluated the effect of hybrid doping Ce and Mn on energy storage efficiency of BaTiO_3_ ceramics. They reported that the Ce and Mn elemental doping promoted the efficiency of energy storage from 65 to 88%.

Moreover, earth-abundant Al is another valuable element as a dopant that enhances the electrochemical properties of the perovskite-based electrocatalysts owing to the creation of oxygen vacancies and modification of electronic structure^22^. Recent studies demonstrated the improvement of the electrochemical performance by doping perovskites with a small percentage of tri-valent Al^3+^ cations^23^. Moreover, based on the former studies, the Al elemental doping in the B-sites of perovskites facilitated the formation of surface oxygen vacancies and oxygen migration from the surface to subsurface^14^. For example, Leonid L. Rusevich et al., fabricated the Al-doped SrTiO_3_ nanoparticles as a photostimulated water Splitting electrocatalyst. They demonstrated that the Al dopant SrTiO_3_ electrode can increase the H_2_ evolution more than 15 times compared with the bare SrTiO_3_ electrode in the water-splitting processes^24^.

According to the literature review, Ce and Al, as nontoxic and low-cost elements, are the potential doping agents to affect electrochemical performance and structural stability of the perovskite BaTiO_3_^14^. Despite this potential, there has been no research to date on the use of Ce and Al co-doping to improve the performance of BaTiO_3_.To address this gap, four electrodes including BaTiO_3_, Al-doped BaTiO_3_, Ce-doped BaTiO_3_, and Al-Ce co-doped BaTiO_3_ were fabricated via two- hydrothermal method. The prepared materials were characterized using diverse methods such as XRD, FT-IR, Raman, XPS, SEM, TEM, HRTEM, EDX, and dot-mapping. The as-prepared electrodes were evaluated for supercapacitor, HER, OER, and overall water splitting. Interestingly, the highest performance was observed in the Al-Ce co-doped BaTiO_3_ electrode, which demonstrates the efficient impact of the doped elements on the structure of BaTiO_3_. This finding highlights the potential of Ce and Al co-doping as a novel strategy to enhance the electrocatalytic activity of BaTiO_3_ and warrants further investigation in this field.

## Results and discussion

### Structural and morphological characterization

**Figure 1:**
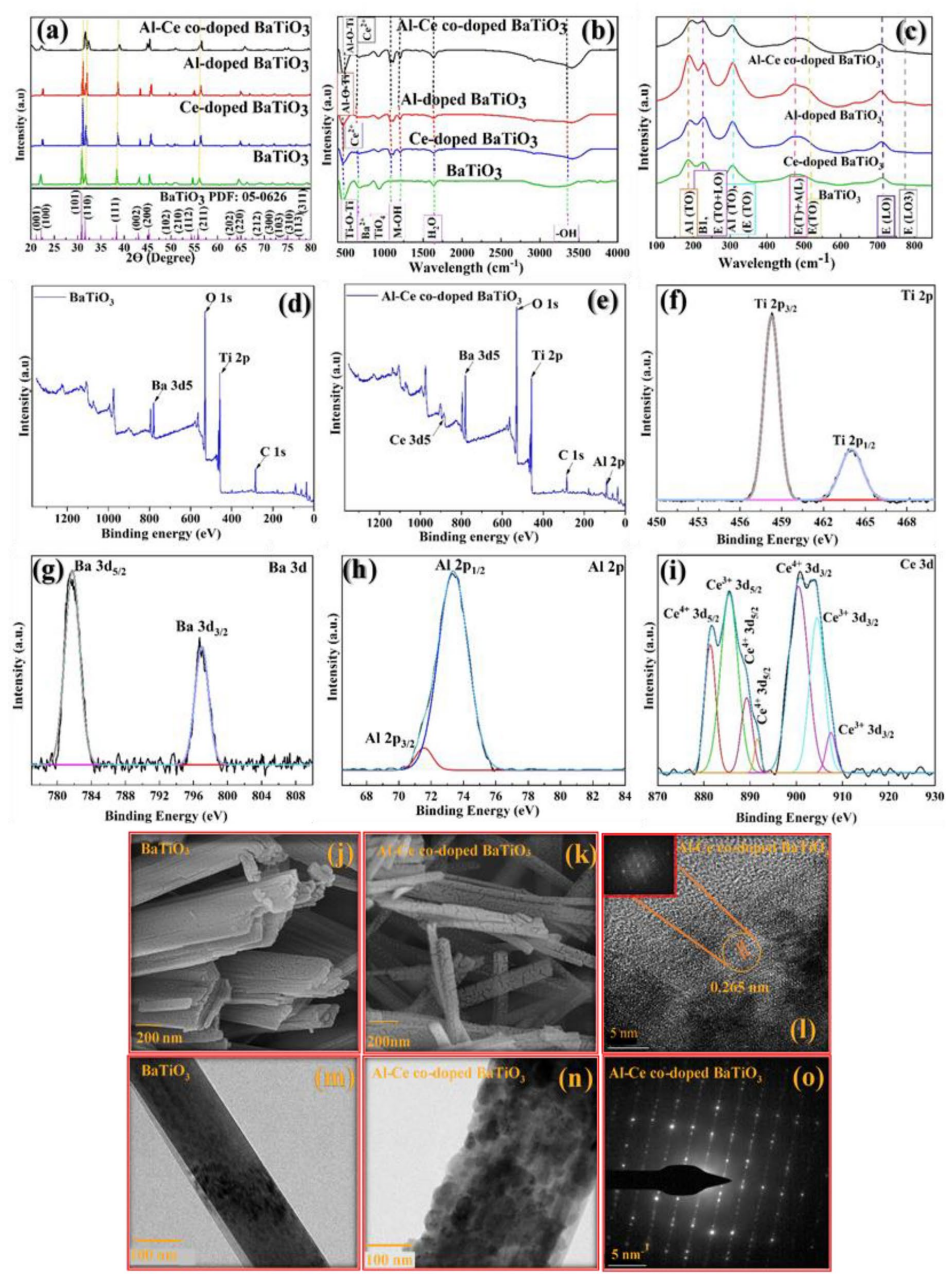
Structural and morphological characterization of the so-synthesized materials. (**a**) The XRD, (**b**) FT-IR and (**c**) Raman spectra of the bare BaTiO_3_, Al-doped BaTiO_3_, Ce**-doped** BaTiO_3,_ and Al-Ce co-doped BaTiO_3_, confirming the successful synthesis of the afore-mentioned nanomaterials. Overall High-resolution XPS survey spectra of bare BaTiO_3_ (**d**) and Al-Ce co-doped BaTiO_3_ (**e**). High-resolution XPS spectra of (**f**) Ti 2p, (**g**) Ba 3d, (**h**) Al 2p, and (**i**) Ce 3d of Al-Ce co-doped BaTiO_3_, demonstrating the contribution of the analyzed functional groups, elements and bonds. (**j**–**k**) The SEM and (**m**–**n**) TEM of BaTiO_3_ and Al-Ce co-doped BaTiO_3,_ respectively. (**l**) HR-TEM image and (**o**) SAED image of Al-Ce co-doped BaTiO_3_, proving the nanofiber configuration and morphology of BaTiO_3_ and Al-Ce co-doped BaTiO_3_ electrocatalysts_._

The crystalline structure of the so-synthesized BaTiO_3_ nanofibers samples were investigated by XRD technique utilizing Cu–Kα (1.5406 Å) radiation at room temperature. According to the XRD pattern shown in Fig. 1a, the main peaks of the pure BaTiO_3_ appeared at 2θ values of 22.0°, 30.9°, 31.3°, 38.4°, 43.0°, 45.4°, 48.4°, 50.1°, 55.1°, 56.1°, 64.8°, and 71.8°, which corresponds to the (100), (101), (110), (111), (002), (200), (102), (201), (211), (211), (210), (202), and (103) planes, respectively (PDF.NO. 05-0626)^25^. Furthermore, the related peaks were also observed for the single and co-doped BaTiO_3_ demonstrating the same crystalline phase and well injection of the dopant elements into the structure of BaTiO_3_. However, in single and co-doped BaTiO_3_ diffractograms the peak positions have nominal right-shifts because of the different radii of doped metals. These minor-right shifts of peaks for doped BaTiO_3_ illustrate the incorporation of doped metals at the interstitial sites of Ti and Ba and lattice shrinkage^26,27^. Indeed, this phenomenon occurs due to the small radius of Al^3+^ (0.535 Å) and Ce^3+^ (1.34 Å) compared to Ba^2+^ (1.61 Å) and Ti^4+^ (0.605 Å). Moreover, keen peaks without any secondary or impurity peaks in diffractograms imply the high purity and the high crystallinity of all samples. In addition, the presence of a peak at 45.4° with the (200) phase plane, proves a tetragonal phase for BaTiO_3_ with space group P4mm (99)^27^.

As part of the investigation into the crystalline structure of the Al-Ce co-doped BaTiO_3_, lattice parameters calculated along the a, b, and c axes and yielded a = b = 3.993442 and c = 4.027835, with 66.89437 Å^3^ unit cell volume. Moreover, the Scherrer equation was utilized for the calculation of crystallite size^28,29^. The value of average crystalline size for Al-Ce co-doped BaTiO_3_ was calculated to be 26.06 nm. Furthermore, the ratio of c/a was 1.050382, denoting the tetragonal structure of Al-Ce co-doped BaTiO_3_ as expected^26^. It is worth noting that, the X-ray diffraction of the as-synthesized Al-Ce co-doped BaTiO_3_ matched with the XRD pattern of the pure BaTiO_3_ and Ce-doped BaTiO_3_^21^, Al-doped BaTiO_3_^25^ and Mn-doped BaTiO_3_^28^ confirmed the incorporation of Al and Ce elements in the BaTiO_3_ lattice.

FT-IR spectra of the samples were reported to evaluate the bonding structure and functional groups of the so-synthesized samples at room temperature. In Fig. 1b, the absorption bands observed at 463, 607, 679, 803, 922, 1630, 2849, 29,914, and 3392 cm^−1^ are present in all samples, indicating their common functional groups and bonding structures. Notably, peaks at 1079, 1118, and 1202 cm^−1^ are exclusively observed in doped samples, confirming the successful incorporation of dopant atoms into the BaTiO_3_ lattice. The absorption band at 463 and 922 cm^−1^ related to the Ti–O–Ti vibration bond and in Al-doped BaTiO_3_ can define as the Al–O–Ti bond^19,30^. Additionally, the peak at 607 cm^−1^ demonstrates the presence of the vibration of metallic cations such as Ba^2+^ in the BaTiO_3_ structure and Ba^2+^ and Ce^2+^ in the Ce-doped BaTiO_3_ structure^31^. Furthermore, the peak at 679 cm^−1^ relates to a stretching bond of Ti–O and Ti–O–Ti and reveals the presence of Ti^4+^ at the (TiO_6_) octahedral units^32^. Moreover, the peak at 679 cm^−1^ was broadened in Ce-doped BaTiO_3_ due to the incorporation of Ce ions, which have a different ionic radius compared to Ba ions, resulting in lattice distortion^26,31^. In addition, the characteristic peak at 803 cm^−1^ proves the presence of the vibration bending of the (TiO_4_) tetrahedral group^32^. In doped samples, new absorbance bands between 1079 and 1202 cm^−1^ confirm the metal-hydroxyl bonding demonstrating the doped metal is successfully bonded to BaTiO_3_^32^. Moreover, the peak at 1630 cm^−1^ depicts the bending vibration of the H–O–H group^29^. Besides, the peaks at 2849, 2914 and 3992 cm^−1^ related to the vibration of O–H groups in the so-synthesized samples^29^.

Using Raman spectroscopy, one can determine the delicate phase, the positions of doped elements, and their effects on the modes. Figure 1c depicts that Ce^2+^ and Al^3+^ have been incorporated into Ti^4+^ and Ba^2+^ sites on co-doped BaTiO_3_. According to the literature, at room temperature, the sharp peaks at 182.7, 228.1, 305.9, 473.5, 535.5, 711.5, 767.7 cm^−1^ are assigned to (A1 (TO)), (B1, E (TO + LO)), (A1 (TO), E (TO)), (E(T) + A(L)), E (TO), (E (LO)), (E (LO3)) Raman modes for pure BaTiO_3_, respectively^32^. Furthermore, in Ce-doped BaTiO_3_ the peak at 187 cm^−1^ shifted to the high wavelength and was determined at 192 cm^−1^, showing the introduction of Ce^2+^ to the Ba^2+^ sites. The Raman spectrum of Ce-doped BaTiO_3_ exhibits a peak at 228 cm^−1^ with the highest intensity, indicating the presence of Ce ions within the BaTiO_3_ lattice^32^. Therefore, the intensity of peaks for Ce-doped BaTiO_3_ increased compared with pure BaTiO_3,_ indicating the incorporation of Ce^2+^ in the lattice of BaTiO_3_. While the peaks in Al-doped BaTiO_3_ do not exhibit significant shifts, the presence of Al ions within the BaTiO_3_ lattice increases peak intensity. In addition, it has a weak shoulder mode at about 473 cm^−1^ depicting the absorption of Al^3+^ in the lattice of BaTiO_3_^19^. Additionally, Al-doped BaTiO_3_ shows increased relative peak intensities compared with pure BaTiO_3_, which can be ascribed to the incorporation of Al^3+^ into the lattice of BaTiO_3_^33^. As a result of the charge imbalance, oxygen vacancies are formed in Al-doped BaTiO_3,_ which is responsible for the peak at 767.7 cm^−1^^34^. Al-Ce co-doped BaTiO_3_ on the other hand contains all the peaks mentioned above that are consistent with the XRD results that demonstrate the doping of metals in the lattice on BaTiO_3_ without any modification in the tetragonal crystalline phase. Our results are similar to those achieved by Liu et al.^21^ and Guo et al.^26^ for Ce-Mn hybrid doped, Al-doped, and Ga-doped BaTiO_3_.

**Figure 2:**
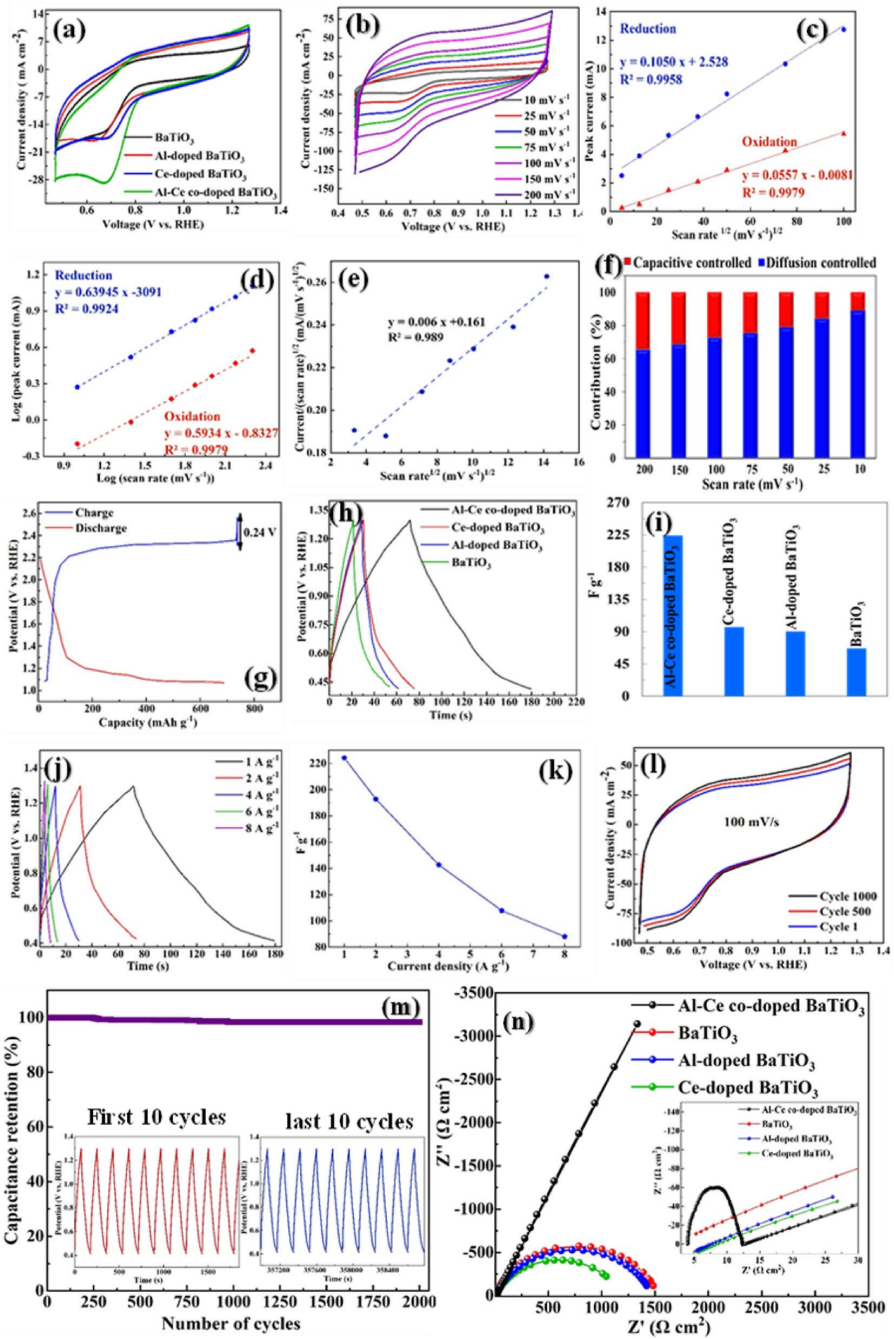
Electrochemical capacitance performance and charge storage properties of the as-prepared electrodes surface. (**a**) Cyclic voltammetry of materials at 10 mV s^−1^ scan rate, verifying the electrocatalytic performance of the Al-Ce co-doped BaTiO_3_ electrode. (**b**) Cyclic voltammetry of Al-Ce co-doped BaTiO_3_ at diverse scan rates from 10 to 200 mV s^−1^, (**c**) Peak current for anodic and cathodic reactions Vs. the square root of the scan rate, confirming the diffusion-controlled processes Al-Ce co-doped BaTiO_3_ electrode. (**d**) b values determined from the linear variation of log (i) and log (υ). (**e**) plot of i(υ)/υ vs υ^1/2^. (**f**) capacitive and diffusion charge contribution of Al-Ce co-doped BaTiO_3_ as function of scan rates. (**g**) Ion transfer kinetics on the surface of the Al-Ce co-doped BaTiO_3_ electrode. (**h**) Galvanostatic charge–discharge curves for so-synthesized materials at 1 A g^−1^ current density, verifying the pseudo-capacitor behavior of the as-prepared electrodes. (**i**) Calculated specific capacitance of materials at 1 A g^−1^ current density from GCD**.** (**j**) Galvanostatic charge–discharge curves at different current densities. (**k**) The calculated specific capacitance for Al-Ce co-doped BaTiO_3_ at diverse current densities from GCD. (**l**) Cyclic stability test of Al-Ce co-doped BaTiO_3_ at 100 mV s^−1^ scan rate, justifying the excellent durability of the Al-Ce co-doped BaTiO_3_ electrode. (**m**) Cycle stabilities of Al-Ce co-doped BaTiO_3_. The insets are GCD curves of the first ten cycles and the last ten cycles of the Al-Ce co-doped BaTiO_3_ during 2000 cycles, respectively. (**n**) Electrochemical impedance (EIS) Nyquist plot of BaTiO_3_, Al-doped BaTiO_3_, Ce-doped BaTiO_3_, and Al-Ce co-doped BaTiO_3_.

The surface elemental states of Al-Ce co-doped BaTiO_3_ NFs investigated by the XPS analysis. The XPS survey spectrum presented in Fig. 1d and e revealed the presence of Ti, Ba, and O elements in BaTiO_3_, and Ti, Ba, O, Al, and Ce elements in Al-Ce co-doped BaTiO_3_. Additionally, Fig. 2f–i and Fig. S1a–d display the high-resolution spectra of these five elements. The binding energies were calibrated for the C 1s peak of graphitic carbon placed at 284.5 eV. As can be seen in Fig. 1f, the two spin–orbit doublet peaks of Ti 2p_3/2_ and Ti 2p_1/2_ are located at 458.3 and 464.0 eV, respectively. Only the peak related to Ti^4+^ was observed for Al-Ce co-doped BaTiO_3_ NFs^35^. Chakrabarti et al.^36^ have also reported the same binding energies for the identification of Ti^4+^ in Cr/BaTiOx/TiN. In addition, as shown in Fig. 1g, the two peaks located at 781.7 and 796.9 eV relate to Ba 3d_5/2_ and Ba 3d_3/2_, respectively^37^. As can be observed in Fig. 1h, the high-resolution spectra of Al revealed the existence of two sub-peaks at 71.5 and 73.4 eV that were assigned to Al 2p_1/2_ and 2p_3/2_ in Al-Ce co-doped BaTiO_3_^38^. Additionally, peaks at 881.4, 889.2, 891.6, and 900.8 eV proved the presence of Ce^4+^, while peaks at 885.5, 903.9, and 907.3 confirmed the existence of Ce^3+^ in Al-Ce co-doped BaTiO_3_ (Fig. 1i). The successful intercalation of dopant elements into the crystalline lattice of BaTiO_3_ was confirmed by the elemental contents presented in Table S1, which align with the expected ratios from the synthesis method, where Al and Ce were added at 3% of Ti and Ba.

According to the previous literature, the perovskite and metal oxides indicate oxygen vacancies^39,40^. Furthermore, the three main Gaussian peaks of the O 1s at 530.0, 530.7, and 532.4 eV indicate the oxygen vacancies of the electrocatalyst samples. Meanwhile, these three peaks illustrated the oxygen of the Ti–O lattice (OL), the evolution of oxygen vacancies (OV), and the adsorption of oxygen species on the electrocatalyst surface (OS), respectively. Besides, Fig. S1a–d exhibits the O 1s spectra of BaTiO_3,_ Al-doped BaTiO_3,_ Ce-doped BaTiO_3_, and Al-Ce co-doped BaTiO_3_. According to the literature review, the OV/OL ratio can quantify the concentration of oxygen vacancies^19^. In this regard, OV/OL for O 1s spectra was 0.317, whereas for BaTiO_3_, Ce-doped BaTiO_3_, and Al-doped BaTiO_3_ were 0.239, 0.245, and 0.280. Moreover, the higher concentration of oxygen vacancies in the Al-Ce co-doped BaTiO_3_ compared to other electrode samples implies that the Al and Ce doping at the Ti and Ba sites improves catalytic activity and electronic conductivity^41^.

Scanning electron microscopy (SEM) and transmission electron microscopy technique (TEM) were used for more investigation of the configuration and morphology of BaTiO_3_, Al-doped BaTiO_3_, Ce-doped BaTiO_3_, and Al-Ce co-doped BaTiO_3_ electrocatalysts. The scanning electron microscope (SEM) images presented in Fig. 1j–k and Fig. S2a, b provide visual evidence of the straight cylindrical shape of the pure BaTiO_3_, Al-doped BaTiO_3_, Ce-doped BaTiO_3_, and Al-Ce co-doped BaTiO_3_. These images show that the samples have a random orientation, indicating that the dopants did not significantly alter the crystal structure of BaTiO_3_. This is an important observation because the crystal structure of a material can greatly affects its properties and behavior, and the fact that the dopants did not disrupt the crystal structure suggests that they were well incorporated into the BaTiO_3_ lattice. As shown in Fig. 1j–k and Fig. S2a, b the average diameter of the bare BaTiO_3_, Al-doped BaTiO_3_, Ce-doped BaTiO_3_, and Al-Ce co-doped BaTiO_3 3_ are 201.15, 85.93, 87.59 and 36.4 nm, respectively. The descending size of the average diameter can be attributed to the substitution of a small ionic radius of Al^3+^ (0.535 Å) instead of Ti^4+^ (0.605 Å) and Ce^3+^ (1.34 Å) instead of Ba^2+^ (1.61 Å)^21^. Furthermore, TEM images (Fig. 1m, n and Fig. S2c, d) depicted that all the samples including BaTiO_3_, Al-doped BaTiO_3_, Ce-doped BaTiO_3_, and Al-Ce co-doped BaTiO_3_ are nanofibers. Additionally, the TEM images depict that the surface morphology of the bare BaTiO_3_ is smooth whereas the surface roughness increased for the Al-doped BaTiO_3_, Ce-doped BaTiO_3_ and Al-Ce co-doped BaTiO_3_ samples. Hence, the obtained results from SEM and TEM images prove the successful introduction of Ce and Al ions into the nanostructure of BaTiO_3_. Delicate evaluation of the crystalline structure of the Al-Ce co-doped BaTiO_3_ nanostructures was carried out by considering high-resolution TEM illustrated in Fig. 1l. Accordingly, the d-spacing of Al-Ce co-doped BaTiO_3_ was calculated to be 0.265 nm which is well-indexed with the (110) plane of the XRD pattern^42^. In addition to the SEM and TEM images, a selected area electron diffraction (SAED) image (Fig. 1o) was obtained to further analyze the crystal structure of the BaTiO_3_ and doped BaTiO_3_ samples. The SAED image showed distinct diffraction spots, which confirm the tetragonal crystalline shape of the samples. The diffraction pattern matches well with the space group P4mm (No. 99) in the International Tables for crystallography, which is consistent with the known crystal structure of BaTiO_3_^43^. For more investigation about the composition of Al-Ce co-doped BaTiO_3_ the EDX and TEM-mapping were accomplished (Fig. S2f–k). Based on the elemental mapping results, the existence of the Al, Ce, Ba, Ti and O elements can be proved in the X-ray emissions^21^.

### Supercapacitor

Cyclic voltammetry (CV) analysis was carried out for the graphite that covered by the BaTiO_3_, Al-doped BaTiO_3_, Ce-doped BaTiO_3_, and Al-Ce co-doped BaTiO_3_ with a three-electrode connection in 1 M KOH to analyze the charge storage on the electrode surface and electrochemical performance. As shown in Fig. 2a, CV curves for the electroactive so-synthesized materials indicate pronounced anodic–cathodic peaks caused by the adsorption of the electrolyte on the surface of electrodes and interaction between the electrolyte and ionic species of electrode (Ba and Ti ions in all so-synthesized electrodes, Al and Ce ions in doped electrodes) during the reversible CV process. This phenomenon demonstrates the pseudo-capacitor behavior of the as-prepared electrodes. Based on the literature review, the pseudo-capacitors overcome the deficiency of the double-layer capacitors. Pseudo-capacitors are able to keep a higher charge than double-layer capacitors due to the store charge through both faradaic and non-faradic processes. In addition, the BaTiO_3_, Ce-doped BaTiO_3_ and Al-doped BaTiO_3_ electrodes display the almost same reduction peak about the − 15.90, − 17.23 and − 18.07 mA, respectively. However, the cathodic peak for the Al-Ce co-doped BaTiO_3_ notably increased to the − 28.94 mA cm^−2^ at 0.67 V vs. RHE. Moreover, the oxidation peak in the BaTiO_3_, Al-doped BaTiO_3_, Ce-doped BaTiO_3_, and Al-Ce co-doped BaTiO_3_ electrodes exhibit the 4.04 to 6.44, 6.95 and 7.21 mA cm^−2^ at 1.027 V vs. RHE, respectively. Hence, introducing Al^3+^, Ce^3+^, and Ce^4+^ metal ions into the BaTiO_3_ lattice notably promoted the cathodic current and slightly the anodic current activity and capacitance behavior of Al-Ce co-doped BaTiO_3_ compared to the bare and single doped of the BaTiO_3_^44^_._

To further investigate the capacitance property and kinetic reaction of Al-Ce co-doped BaTiO_3_, we conducted CV measurements using seven different scan rates ranging from 10 to 200 mV s^−1^. When the scan rate is increased, the ions in the electrolyte do not have sufficient time to diffuse into the surface of the host sites. As a result, the specific capacitance decreases. Nevertheless, due to the current is proportional to the scan rate, the current density increase. Additionally, the active species cannot sustain intercalation on the electrode surface, leading to a decrease in the specific capacitance at high scan rates. As expected, despite the reduction in specific capacitance, the increased current density with higher scan rates demonstrates the high-rate capability of the prepared electrode^16^. Figure 2b demonstrates a significant increase in current density as the scan rate varied from 10 to 200 mV s^−1^ at 0.67 V vs. RHE, leading to values of 26.30, 35.62, 55.02, 72.17, 87.94, 116.27, and 142.02 mA cm^−2^. Furthermore, at higher scan rates, the peak positions slightly shifted due to electrode polarization and efficient kinetic reactions taking place on the Al-Ce co-doped BaTiO_3_ electrode surface.

The data presented in Fig. 2c shows that the current density peak for both anodic and cathodic reactions (mA) is proportional to the square root of the scan rate, indicating that the redox reaction occurring on the electrode surface is a diffusion-controlled process. Furthermore, the presence of doped BaTiO_3_ in the system does not appear to impede the capacitance process, indicating that it does not act as a restriction factor. These findings suggest that the addition of doped BaTiO_3_ may have beneficial effects on the electrochemical performance of the system without negatively impacting its capacitive behavior. For more investigation into the charge storage mechanism of the Al-Ce co-doped BaTiO_3_ electrode, the power law equation is applied (Eq. 1).

1 $${\text{i }} = \, \alpha \upsilon^{{\text{b}}}$$

where i represents the peak current values (mA), υ indicates scan rates (mV s^−1^), α and b refer to empirical parameters. In this law, b value close to 0.5 signifies the dominating diffusion controlled faradic process whereas b value near 1 means the capacitive controlled process^45^ (Fig. 2d). The b values of anodic and cathodic currents for the Al-Ce co-doped BaTiO_3_ are close to 0.5 (0.59 and 0.63), implying the diffusion controlled reversible process of the electrode. Furthermore, to explore the charge storage contribution mechanisms of Al-Ce co-doped BaTiO_3_ electrode at different scan rates, the capacitive (K_1_υ) and diffusion (K_2_ υ ^1/2^) contribution can be quantified according to the Eq. (2).

2 $${\text{i}}\left( \upsilon \right) \, = {\text{ K}}_{{1}} \upsilon \, + {\text{ K}}_{{2}} \upsilon^{{{1}/{2}}}$$

where, i(υ) and υ are the current response and scan rates, respectively^46^ (Fig. 2e). In Fig. 2f, it can be witnessed that the charge storage mechanism of Al-Ce co-doped BaTiO_3_ is governed by diffusion-controlled reaction at high scan rates (95% at 10 mV s^−1^) and since the sectional intercalation of ions at lower scan rates, the capacitive contribution increases gradually. Furthermore, the presence of doped BaTiO_3_ in the system does not appear to impede the capacitance process, indicating that it does not act as a restriction factor. These findings suggest that the addition of doped BaTiO_3_ may have beneficial for ion diffusion in the electrode materials, facilitating an ultra-fast charge discharge process and improving electrochemical performance of the system without negatively impacting its capacitive behavior.

The voltage profile for Al-Ce co-doped BaTiO_3_ electrode is shown in Fig. 2g. The maximum charging voltage for Al-Ce co-doped BaTiO_3_ electrode was calculated to be 1.49 V. In order to measure the kinetics of ion transfer on the surface of the electrode, the GITT technique was used. In this technique, first, the closed-circuit voltage (CCV) was measured. When the voltage reached its maximum value in a short period of time, the quasi-open circuit voltage (QOCV) discharge was measured. The QOCV value for the Al-Ce co-doped BaTiO_3_ was observed as 1.25 V and its difference from CCV was calculated as 0.24 V. The difference between the voltage of QOCV and CCV is related to the diffusion of electrolyte ions, and the reduction of this value indicates the piezoelectric property of the Al-Ce co-doped BaTiO_3_^47^. In addition, the coulombic efficiency was calculated from the derivative of the CV current density for the Al-Ce co-doped BaTiO_3_ electrode. The coulombic efficiency of the Al-Ce co-doped BaTiO_3_ was obtained 100%, which reveals the high electroactivity performance of the active materials and the complete diffusion of the electrolyte on the electrode surface^48^.

Three-electrode galvanostatic charge–discharge (GCD) systems can demonstrate the same tendency. As can be seen in Fig. 2h, the Al-Ce co-doped BaTiO_3_ performs a better discharge time than the bare and single-doped BaTiO_3_ electrodes. Moreover, variation of the slopes for the charge–discharge curves can be observed due to the redox activity of electrode ion species verifying the pseudo-capacitor behavior of the as-prepared electrodes. In addition, based on the charge–discharge duration, the mass-specific capacitance (Cp) computed at 1 A g^-1^ current by utilizing the following formula^49^, where I, $\Delta$ t, m, and $\Delta$ V are the current density (A), time (s), mass (g), and applied voltage (v), respectively.

3 $${\text{Cp }}\left( {{\text{F}}\;{\text{g}}^{{ - {1}}} } \right) \, = \frac{{{\text{I }}\Delta {\text{t}}}}{{{\text{m }}\Delta {\text{V }}}}$$

According to the obtained results in Fig. 2i, the Cp amount of the Al-Ce co-doped BaTiO_3_ is 224.18 F g^−1^, which is the greatest one compared to the BaTiO_3_, Al-doped BaTiO_3_, and Ce-doped BaTiO_3_ electrodes (66.6, 90.3 and 96.4 F g^−1^, respectively). The specific capacitance of this study compared to previously published supercapacitors made with perovskite-based electrocatalysts and results are shown in Table 1. The Al-Ce co-doped BaTiO_3_ indicated a significantly higher specific capacitance in comparison to previous research findings. The Table 1 also demonstrates that we utilized a simple, readily available, low-cost experimental situation such as low concentration of NaOH as an electrolyte (1 M), low scan rate (10 mv s^−1^), economic electrocatalysts, and graphite as an electrocatalyst bed.

**Table 1:** Electrochemical parameters for current study and previously published supercapacitors made with perovskite-based electrocatalysts.

Row	Electrocatalyst	Electrolyte		Scan rate (mVs^−1^)	Potential range (V vs. RHE)	Specific capacitance (Fg^-1^)	Refs.
1	BaTiO_3_	1 M KOH		10	0.7–1.3	66.6	This study
2	Ce-doped BaTiO_3_	1 M KOH		10	0.7–1.3	96.4	This study
3	Al-doped BaTiO_3_	1 M KOH		10	0.7–1.3	90.3	This study
4	Al-Ce co-doped BaTiO_3_	1 M KOH		10	0.7–1.3	224.18	This study
5	Sr-and Fe-substituted LaMnO_3_	3 M KOH		20	0.82–1.32	255	^54^
6	BiFeO_3_- BaTiO_3_ composite	PVA/H_3_PO_4_ gel		50	0–1.2	150	^55^
7	SrTiO_3_	1 M KCl		1	(− 0.3)–0.3	208.47	^56^
8	B-Doped SrTiO_3_/Ni Sheet	0.5 NaOH		60	0.3–0.5	5.488	^57^
9	Polyaniline-SrTiO_3_ nanocube	1 M KOH		5	0.82–1.32	127.5	^58^
10	SrTiO_3_	3 M KOH		5	0.62–1.42	212.5	^59^
11	SrMnO_3_	0.5 M Na_2_SO_4_		10	0.82–1.62	176.2	^11^
12	LaNiO_3_	1 M Na_2_SO_4_		10	0.82–1.82	160	^69^

In addition, the charge–discharge test at different current densities ranging from 1 to 8 A g^−1^ were performed for more examination of the electrochemical performance of Al-Ce co-doped BaTiO_3_ in Fig. 2j. All charge–discharge curves prove the superlative coulombic efficiency and high faradic redox reaction due to the almost symmetric and linear profile in Al-Ce co-doped BaTiO_3_^16^. As can be observed in Fig. 2k, the Cp at diverse current densities was determined as 224.18, 192.77, 142.72, 107.90, and 88.00 F g^−1^ at 1, 2, 4, 6, and 8 A g^−1^, respectively. Therefore, the simultaneous incorporation of Al and Ce dopants in the crystalline lattice of the BaTiO_3_ significantly developed its electrochemical capacitive performance owing to the enhancing oxygen vacancy and redox activity^44^.

To study the durability of the Al-Ce co-doped BaTiO_3_ electrode, long-term CV was conducted at 100 mV s^−1^ scan rate. As can be observed in Fig. 2l, the stability of the Al-Ce co-doped BaTiO_3_ electrode after 1000 cycles showed no significant change, which indicates the excellent durability of the electrode due to the fast electron transfer among the reaction sites of the Al-Ce co-doped BaTiO_3_ electrode and high electrolyte diffusion^16^. In a study conducted by Ben Cheikh et al.^50^ the supercapacitor properties of hydrogen-doped BaTiO_3_ films were investigated. They found that the hydrogenated barium titanate films exhibited better capacitance features, including a large potential window, a wider operational temperature range, proper stability after 400 CV cycles, and faster charge/discharge cycles, compared to the bare BaTiO_3_ films. In addition, the Charge–discharge cycles were carried out to evaluate the stability of the Al-Ce co-doped BaTiO_3_ electrocatalyst at 1 A g^−1^ and the obtained results are shown in Fig. 2m. According to the results, the cell can retain 98% of initial capacitance over the 980 cycles and it maintains a steady capacitance for the rest of the sweeping cycles. The inset pictures of Fig. 2m display the initial and final ten charge/discharge cycles, demonstrating no change in response shape^51,52^. The attained outcomes well agreed with each other and confirm the excellent stability of the Al-Ce co-doped BaTiO_3_ electrocatalyst.

To support the previous results, electrochemical impedance spectroscopy (EIS) was applied for all as-prepared samples and fitted Nyquist plot portrayed from low to high frequencies in Fig. 2n with negligible resistance between the electroactive materials in the electrode and electrolyte (R_s_) (~ 4 Ω). As can be observed in the Nyquist plot, the diameter of the BaTiO_3_ semicircle has been decreased from 1484 to 1400 Ω cm^2^ and 1043 Ω cm^2^ for the Al-doped BaTiO_3_ and Ce-doped BaTiO_3_. This phenomenon indicates the incorporation of dopants within the electrodes and reduction of charge transfer resistance (R_ct_) owing to the improved conductivity. Furthermore, the Warburg Nyquist curve of the Al-Ce co-doped BaTiO_3_ with ignorable semicircle elucidating faradic redox reaction, the fast ion diffusion, and perfect capacitive behavior coupled with the scant amount of the charge transfer resistance (R_ct_). In addition, the large angle between the Z’ and the Warburg curve of the Al-Ce co-doped BaTiO_3_ reiterates the pseudo capacitance behavior without diffusion limitation of asymmetry supercapacitor. The electrochemical mechanism of the Al-Ce co-doped BaTiO_3_ electrode can be defined in three steps. First, OH was generated in the electrolyte from the oxygen. Second, the formed OH groups fill the oxygen vacancies and they oxidate the Ce and Al in the Al-Ce co-doped BaTiO_3_ along the crystalline tetrahedral edges owing to their high oxidation activity. Third, during the charge and discharge cycles of a pseudo-capacitor, extra oxygen atoms can intercalate at the surface of the Al-Ce co-doped BaTiO_3_ electrode. This process occurs through the diffusion of Ba and Ce elements at the surface of the electrode, as well as the oxidation of Ba and Ce ions in the center of the perovskite structure. Compared to metal oxides, O_2_ and metal oxides can carry out two negative charges per unit and one negative charge per unit, respectively. Therefore, in each cycle of charge/discharge, O_2_ can retain twice the negative charge than metal oxides. Consequently, the Al and Ce dopant can improve the capacitance properties of Al-Ce co-doped BaTiO_3_ electrode owing to the creation of deficient at the crystalline structure of BaTiO_3_, improvement of oxygen vacancy and charge transfer rate in the electrode, and increased electrocatalytic ability^11,53^.

### OER and HER

**Figure 3:**
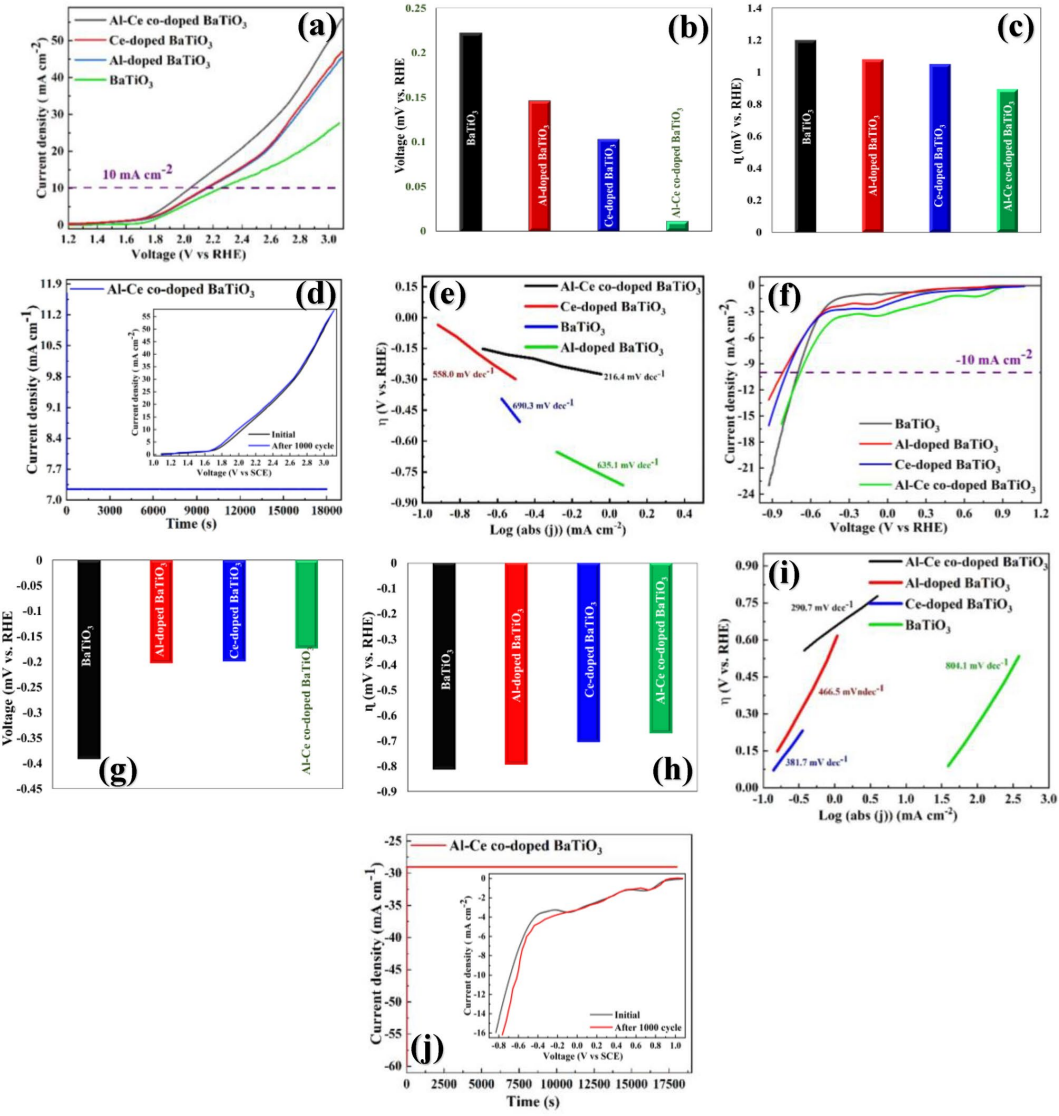
Electrocatalytic OER and HER activity in KOH solution (1 M). (**a**) OER polarization curves (**b**) on-set potential (**c**) overpotential, and (**d**) Tafel slopes of BaTiO_3_, Al-doped BaTiO_3_, Ce-doped BaTiO_3_, and Al-Ce co-doped BaTiO_3_ at 10 mV s^−1^, showing the superlative OER and HER performance of the Al-Ce co-doped BaTiO_3_ electrode compared to so-synthesized electrodes. (**e**) Chronoamperometry and stability tests with an inserted figure showing LSV polarization curves for Al-Ce co-doped BaTiO_3_ before and after 1000 CV cycles, confirming the durable efficiency of the Al-Ce co-doped BaTiO_3_ electrode compared to as-prepared electrodes. (**f**) HER polarization curves (**g**) on-set potential (**h**) overpotential, and (**i**) Tafel slopes of BaTiO_3_, Al-doped BaTiO_3_, Ce-doped BaTiO_3_, and Al-Ce co-doped BaTiO_3_ at 10 mV s^−1^, depicting the superlative HER and HER performance of the Al-Ce co-doped BaTiO_3_ electrode compared to so-synthesized electrodes. (**j**) Chronoamperometry and stability tests with an inserted figure showing LSV polarization curves for Al-Ce co-doped BaTiO_3_ before and after 1000 CV cycles confirming the durable efficiency of the Al-Ce co-doped BaTiO_3_ electrode compared to as-prepared electrodes.

For more investigation about the electrochemical performance of BaTiO_3_, Al-doped BaTiO_3_, Ce-doped BaTiO_3_, and Al-Ce co-doped BaTiO_3_, the linear sweep voltammetry (LSV) was conducted in a 1.0 M KOH electrolyte utilizing the standard three-electrode system. Figure 3a depicts the polarization curve of the as-prepared electrodes at the scan rate of 10 mV s^−1^ for oxygen evolution reaction (OER) under the O_2_-saturated condition. Based on the CV curve in Fig. 2a oxidation peak in Al-doped BaTiO_3_, Ce-doped BaTiO_3_ and Al-Ce co-doped BaTiO_3_ intensified and indicated a slight right side-shifted. As expected, in Fig. 3a, the LSV curve of the BaTiO_3_ electrode shows the rightward shift after doping by Al and Ce elements and improving the OER property of the bare BaTiO_3_. As can be seen in Fig. 3b, the onset potential of Al-Ce co-doped BaTiO_3_ (11 mV vs. RHE) is much lower than BaTiO_3_ (222 mV vs. RHE), Ce-doped BaTiO_3_ (103 mV vs. RHE) and Al-doped BaTiO_3_ (146 mV vs. RHE). In Fig. 3c, the co-doped BaTiO_3_ sample shows a significantly lower overpotential of 894 mV vs. RHE compared to bare BaTiO_3_ (1200 mV vs. RHE), Al-doped BaTiO_3_ (1080 mV vs. RHE), and Ce-doped BaTiO_3_ (1050 mV vs. RHE). These results suggest that the co-doping of Al and Ce can greatly enhance the electrocatalytic activity of the BaTiO_3_ electrode, likely due to an increase in surface conductivity and charge transfer. Therefore, the simultaneous incorporation of Al and Ce is a promising strategy for improving the performance of BaTiO_3_ as an electrocatalyst. In addition, the mechanism and kinetics of the as-prepared electrodes for OER were investigated by the Tafel slope driven from LSV curves as illustrated in Fig. 3d. The co-doped BaTiO_3_ electrode indicates the 290 mV dec^−1^ which is lower than BaTiO_3_ (804.1 mV dec^−1^), Ce-doped BaTiO_3_ (381.7 mV dec^−1^) and Al-doped BaTiO_3_ (466.5 mV dec^−1^) improve the positive influence of the dopant on the kinetic of the water oxidation of the as-synthesized electrodes. Furthermore, the durability of the co-doped electrode was examined by 5 h chronoamperometry and after that, 800 cycles OER continuously. As can be seen in Fig. 3e, the Al-Ce co-doped BaTiO_3_ displays outstanding electrocatalytic stability during these processes.

Moreover, to the great OER activities, the Al-Ce co-doped BaTiO_3_ electrode shows superior hydrogen evolution reaction (HER) performance. It is worth noting that, in the CV diagram in Fig. 2a, the Al-doped BaTiO_3_ reduction peak intensified, and the Ce-doped BaTiO_3_ reduction peak shifted toward the positive voltage. Moreover, the Al-Ce co-doped BaTiO_3_ electrode indicates the both properties of Al-doped and Ce-doped BaTiO_3,_ simultaneously. As can be observed in Fig. 3f, the Al-Ce co-doped BaTiO_3_ electrode displays the best electrocatalytic HER performance compared to the other as-prepared electrodes. The broad cathodic peak was found at around 0.8 and − 0.02 V vs. RHE probably related to the reduction of Ce^4+^ and Al^3+^. Besides, as can be seen in Fig. 3f, the HER activity of the Al-Ce co-doped BaTiO_3_ is lower than pristine BaTiO_3_ at higher current densities (more than 13 mA cm^−2^). The reason for this phenomenon may be due to the phase-modification strategies that can control the local structure of surface adsorption sites through the crystallinity and Miller index of catalyst crystal planes, which is of great significance in determining the HER catalytic behavior^60^. According to the previous literature, the (111) plane exhibited better HER activity in the high-overpotential region, whereas the opposite was true in the low-overpotential region^61^. In addition, based on the Fig. 1a, the (111) phase plane in XRD pattern of bare BaTiO_3_ is sharper than the Al-Ce co-doped BaTiO_3._ For instance, the Tao Wu et al.^61^ reported the weak HER performance at low-overpotential and high HER activity at high-overpotential for the (111) phase plane of the CoPS compared to other phase planes of the CoPS. Furthermore, Fig. 3g presents the onset potential of the as-prepared electrodes. The co-doped BaTiO_3_ indicates a smaller onset potential (− 174 mV vs. RHE) than the BaTiO_3_ (− 391 mV), Ce-doped BaTiO_3_ (− 199 mV) and Al-doped BaTiO_3_ (− 203 mV). Besides, the overpotential of co-doped BaTiO_3_ is − 570 mV vs. RHE, whereas the bare, Ce-doped and Al-doped electrodes show − 705, − 794, and − 812 mV vs. RHE, respectively (Fig. 3h). Additionally, Fig. 3i depicts the Tafel plots extracted from HER polarization curves. According to the Tafel slopes, the Al-Ce co-doped BaTiO_3_ illustrates the smallest slope among the other prepared electrodes and its amount is 216 mV dec^−1^. However, the Tafel slope for bare, Al-doped and Ce-doped is 690, 635 and 558 mV dec^−1^. For more investigation about the stability of Al-Ce co-doped BaTiO_3_ the 5 h chronoamperometry and after that, 800 cycles HER were accomplished continuously (Fig. 3j). Based on the chronoamperometry results, the co-doped BaTiO_3_ electrode indicates excellent durability during the 5 h without any reducing the current density. On the other hand, the conducted HER analysis also depicts the great stability during 800 cycles. Hence, the co-doped BaTiO_3_ electrode possesses the appropriate OER and HER performance serving as a promising electrocatalyst for oxygen and hydrogen evolution reactions. This high electrochemical performance can demonstrate high speed charge transfer, enhance conductivity and increase the generation of oxygen vacancies due to the doping of Al and Ce elements. The above-mentioned results shown in Table 2. In addition, the OER and HER performance of so-synthesized electrodes compared to previously published OER and HER efficiency made with perovskite-based electrocatalysts and results are shown in Table 3. In order to evaluate the electrocatalytic active surface area (ECSA) of BaTiO_3_, Al-doped BaTiO_3_, Ce-doped BaTiO_3_, and Al-Ce co-doped BaTiO_3_ electrocatalysts, the charging current measurement carried out at non-faradaic region (0.95 V vs. RHE) of CV diagram, by the Eqs. (4) and (5).

4 $${\text{ECSA}}_{{}} \left( {{\text{cm}}^{{2}} } \right) \, = \frac{{{\text{Cdl}}}}{{{\text{Cs}}}} \times {\text{ geometrical surface}}$$

5 $${\text{C}}_{{{\text{dl}}}} \left( {{\text{mF cm}}^{{ - {2}}} } \right) \, = \frac{{{\text{Ja}} - {\text{Jc}}}}{{2\left( {\text{scan rate}} \right)}}$$

which ECSA, C_s_, C_dl_, Ja, Jc and scan rate are electrocatalytic active surface area (cm^2^), capacitance of the flat electrode surface (0.04 mF cm^−2^), double-layer capacitance (mF cm^−2^), anodic current density (mA cm^−2^), cathodic current density (mA cm^−2^), and applied scan rate (V s^−1^), respectively^41^. Accordingly, the C_dl_ for BaTiO_3_, Al-doped BaTiO_3_, Ce-doped BaTiO_3_, and Al-Ce co-doped BaTiO_3_ electrocatalysts calculated 310, 485, 500 and 533 mF cm^−2^, respectively. In addition, the ECSA amounts of afore-mentioned electrocatalysts calculated 7440, 11,640, 12,000, and 12,797 cm^2^, respectively. From the above results, the relative electrochemical surface area of Al-Ce co-doped BaTiO_3_ is 1.71 times of BaTiO_3_. The active electrochemical surface area of investigation also supports that Al-Ce co-doped BaTiO_3_ increases the active sites, which stimulate greater OH exposure to improve the overall electrical conductivity from the water OH radicals and functional electrode surfaces. Consequently, the more active sites enhance the OER and HER efficiency.

**Table 2:** O_2_ and H_2_ evolution of Al-Ce co-doped BaTiO_3_ electrode.

Electrocatalytic activity	Electrode	Onset potential (V vs. RHE)	Over potential (V vs. RHE) at 10 mA cm^−2^	Tafel slope (mV dec^−1^)
OER	BaTiO_3_	0.222	1.20	804.1
	Al-doped BaTiO_3_	0.146	1.08	466.5
	Ce-doped BaTiO_3_	0.103	1.05	381.7
	Al-Ce co-doped BaTiO_3_	0.011	0.894	290
HER	BaTiO_3_	− 0.391	− 0.812	630
	Al-doped BaTiO_3_	− 0.203	− 0.794	635
	Ce-doped BaTiO_3_	− 0.199	− 0.705	558
	Al-Ce co-doped BaTiO_3_	− 0.174	− 0.570	211

Electrocatalytic OER and HER activity in KOH solution (1 M) at 10 mV s^−1^, indicating the superlative OER and HER performance of the Al-Ce co-doped BaTiO_3_ electrode compared to so-synthesized electrodes.

**Table 3:** Electrochemical parameters for Current study and previously published water splitting made with perovskite-based electrocatalysts at 10 mAcm^−2^ Current density.

Row	Electrocatalyst	Electrolyte	OER overpotential range (V vs. RHE)	HER overpotential range (V vs. RHE)	Refs.
1	BaTiO_3_	1 M KOH	1.20	− 0.812	This study
2	Ce-doped BaTiO_3_	1 M KOH	1.08	− 0.794	This study
3	Al-doped BaTiO_3_	1 M KOH	1.05	− 0.705	This study
4	Al-Ce co-doped BaTiO_3_	1 M KOH	0.894	− 0.570	This study
5	IrO_2_	1 M KOH	0.44	–	^63^
6	[PrBa_0.5_Sr_0.5_]_0.95_Co_1.5_Fe_0.5_O_5+d_, P-HF	1 M KOH	0.53	–	^64^
7	La_0.5_(Ba_0.4_Sr_0.4_Ca_0.2_)_0.5_Co_0.8_Fe_0.2_O_3–δ_ /rGO	1 M KOH	0.338	− 0.235	^64^
8	NaNbO_3_	–	0.90	− 0.6	^65^
9	SrNb _0.1_ C_0.7_Co_1.5_Fe_0.2_O_3+δ_	0.1 M KOH	0.39	0.262	^66^
10	Ca_2_FeRuO_6_	1 M KOH	0.4	0.42	^67^
11	RuO_2_	1 M KOH	0.29	–	^67^
12	Pt/C	1 M KOH	–	0.05	^67^
13	Sr_0.95_Nb_0.1_Co_0.9_ − xNi_x_O_3-δ_	1 M KOH	0.38	− 0.299	^68^
14	(PrBa_0.5_Sr_0.5_)_0.95_Co_1.5_Fe_0.5_O_5+δ_	0.1 M KOH	0.32	− 0.23	^63^
15	SrIr_0_._33_Ti_0.6_7O_3_	–	0.247	–	^69^
16	CaCu_3_Ru4O_12_	0.5 M H_2_SO_4_	0.171	–	^69^

Turnover frequency (TOF) is another intrinsic activity parameter that could be derived from that current density at a fixed potential and the surface concentration or number of actually involved metal sites. The turnover frequency (TOF) was evaluated by the following Eq. (6).

6 $${\text{TOF }}\left( {{\text{s}}^{{ - {1}}} } \right) \, = \frac{{\text{I}}}{{4{\text{Fm}}}}$$

where I, F, and m are the current (A), Faraday constant (96,485.3321 s A mol^−1^), and the number of moles in the catalyst, respectively^62^. The TOFs of OER for BaTiO_3_, Al-doped BaTiO_3_, Ce-doped BaTiO_3_, and Al-Ce co-doped BaTiO_3_ electrocatalysts were 7.06 × 10^–1^, 5.78 × 10^–1^, 2.54 × 10^–2^ and 3.017 × 10^–2^ s^−1^ at an overpotential of 2.07 V vs. RHE, respectively. In addition, The TOFs of HER for BaTiO_3_, Al-doped BaTiO_3_, Ce-doped BaTiO_3_, and Al-Ce co-doped BaTiO_3_ electrocatalysts were 5.98 × 10^–1^, 6.30 × 10^–1^, 6.67 × 10^–1^ and 1.55 × 10^–2^ s^−1^ at an overpotential of 0.97 V vs. RHE, respectively. Therefore, the Al-Ce co-doped BaTiO_3_ electrocatalysts indicate the superlative OER and HER performance among the other as-prepared electrocatalysts.

### Overall water splitting

**Figure 4:**
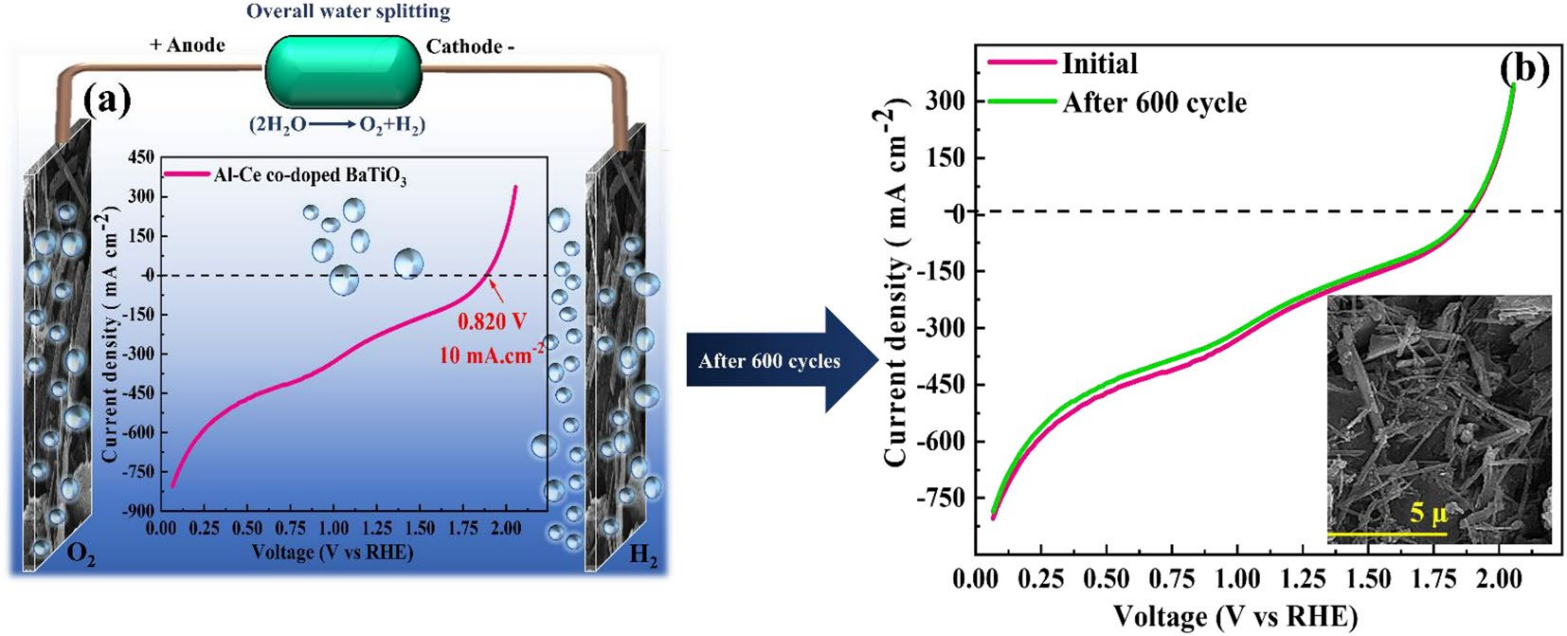
Overall water splitting performance of Al-Ce co-doped BaTiO_3_. (**a**) LSV polarization curve at 10 mA cm^−2^, (**b**) LSV polarization curve for Al-Ce co-doped BaTiO_3_ before and after 600 CV cycles at 10 mA cm^−2^, and the inset shows the SEM of Al-Ce co-doped BaTiO_3_ nanofiber after the stability test, demonstrating the durability and superlative water splitting efficiency of the Al-Ce co-doped BaTiO_3_ electrode.

Owing to the effective OER and HER results for Al-Ce co-doped BaTiO_3_, the water splitting performance was demonstrated by a two-electrode cell. To achieve this purpose, the 46.66 mg Al-Ce co-doped BaTiO_3_ homogeneous ink was loaded on both sides of the two pieces of the 3 × 3 × 0.5 cm graphite with 21 cm^2^ surface area as cathode and anode. These two pieces of electrodes were separated by utilizing the Nafion membrane as ions exchanger. As can be observed in Fig. 4 the LSV at 10 mV s^−1^ scan rate and durability were carried out at different voltages. Based on the obtained results in Fig. 4a, at 10 mA cm^−2^, the overpotential could achieve 0.820 V proving the superlative water splitting performance^70–72^. Parangusan et al.^73^ investigated the Hierarchical BaTiO_3_/NiFe_2_O_4_ nanocomposite as an efficacious photoanode for photoelectrochemical water splitting. They reported that the BaTiO_3_/NiFe_2_O_4_ reached 1.64 V overpotential at 0.35 mA cm^−2^ which demonstrates the low efficiency of the BaTiO_3_/NiFe_2_O_4_ electrocatalyst for water splitting performance. In addition, the stability of the water splitting cell was evaluated by overall LSV during 500 cycles and SEM image before and after 500 cycles overall LSV. Figure 4b illustrates that after 500 cycles no change was observed in the LSV curve, which verifies the good durability of the Al-Ce co-doped BaTiO_3_ electrode in water splitting. Besides, the SEM images of the Al-Ce co-doped BaTiO_3_ electrode before and after 500 cycles indicate no obvious alteration in the morphology of the coated electrocatalyst on the surface of the electrode demonstrating the desirable stability of the Al-Ce co-doped BaTiO_3_.

According to the literature review, the replacement of the Ti ion (0.605 Å) by Al ion (0.535 Å) and Ba ion (1.61 Å) by Ce ion (1.34 Å) can improve the charge transferring. A doping element into the A and B-site with a lower radius compared to the host ion provides more open space, enabling the larger displacement of Ba and Ti, distorts oxygen octahedron surrounding a dopant, decreasing the lattice parameters, destabilizes the tetragonal phase and consequently promotes the electronic features of the BaTiO_3_^74^. For instance, Ahmed et al.^74^ reported the replacement of the Ba^2+^ ion by smaller ions in the A-site of BaTiO_3_ can enhance polarization, which potentially originates from a growth in the size of polarized domain walls and improves domain ordering during the electric poling sequence. Besides, Kang Yan and co-workers^75^ evaluated the effect of simultaneous doping of Ce^3+^-Gd^3+^ elements on B-site of BaTiO_3_. They reported that the elemental doping with a smaller radius in B-site enhances the concentration of oxygen vacancies. In addition, the Zechao Li et al.^76^ the substitution on either the A- or B-site of environmentally-friendly BaTiO_3_ ceramics by elements has a profound influence on their properties and provides a promising opportunity to tune the dielectric, ferroelectric, and piezoelectric properties.

Ce ion is an excellent carrier for oxygen storage and release due to abundant oxygen vacancy derived from the mutual conversion of Ce^3+^/Ce^4+^ redox pairs^77^. According to Eq. (7), Ce^3+^ ions donate extra electrons at the surface of the BaTiO_3,_ leading to the enhancement of the H_2_ evolution reaction rate^19^.

7 $${\text{4H}}_{{2}} {\text{O }} + {\text{ 4e}}^{ - } \to {\text{2H}}_{{2}} + {\text{ 4OH}}^{ - }$$

In addition, many of afore-mentioned advantages of Ce doping arise from the facility of the Ce^3+^/Ce^4+^ redox couple and the high mobility of nonstoichiometric-induced oxygen vacancies in nanosized ceria materials. Briefly, the mechanism of free radical scavenging in surface Ce ions revolves around the multiple oxidation states of the metal ion. In addition, Ce^3+^ is considered to be highly efficient radical scavenger, with a rapid and regenerative redox reaction^78^. The present •OH in solution can be scavenged by lattice oxygen vacancy sites, resulting in the concomitant oxidation of Ce^3+^ to Ce^4+^. Once Ce^3+^ gets oxidized through a reaction with HO•, there is a mechanism for its regeneration (and hence vacancy regeneration) on the surface of ceria nanoparticles. The following reactions have been proposed for free radical scavenging, Ce^3+^ regeneration and oxygen revolution. During free radical scavenging, the surface Ce^3+^ is oxidized to Ce^4+^ as Eq. (8):

8 $$^{ \bullet } {\text{OH }} + {\text{ Ce}}^{{{3} + }} \to {\text{Ce}}^{{{4} + }} + {\text{ H}}_{{2}} {\text{O}}$$

During Ce ions regeneration, surface Ce^4+^ is reduced back to Ce^3+^ on the surface of ceria by the following reaction^77^:

9 $${\text{4Ce}}^{{{4} + }} + {\text{2H}}_{{2}} {\text{O}} \to {\text{4Ce}}^{{{3} + }} + {\text{4H}}^{ + } + {\text{O}}_{{2}}$$

The above phenomena benefit electrocatalytic water-splitting processes and promote the successive generation of H_2_ and O_2_.

Furthermore, the hydrothermal synthesis of BaTiO_3_ involves a two-step process. In the initial step, H_2_Ti_3_O_7_ nanofibers precipitate, subsequently incorporated into a solution of Ba (OH)_2_·8H_2_O for a secondary hydrothermal treatment, ultimately forming BaTiO_3_. Notably, during the first step, an aluminum (Al) doping agent is introduced to the solution, while cerium (Ce) dopants are added in the second step^32^. The anticipation is that Al will substitute for Ti in the B-site, and Ce will substitute for Ba in the A-site. This hypothesis can be further substantiated through the similar radius of Al^3+^ (0.535 Å) with Ti^4+^ (0.605 Å) and Ce^3+^ (1.34 Å) with Ba^2+^ (1.61 Å) and the tetragonal crystallin structure of Al-Ce co-doped BaTiO_3_ which demonstrated in characterization section. Adak et al.^32^ demonstrated the successful substitution of Mn in the B-site and the Ce in the A-site of the BaTiO_3_ using the e tetragonal phase with P4mm space group which supported from XRD, Raman, and EDX analyses.

Moreover, our catalyst is heterogenous and Ce element doped into the crystalline lattice of the BaTiO_3_. Therefore, for more evaluation about the leaching elements into the electrolyte and homogeneous reactions, the atomic absorption spectroscopy (AAS) was assessed after the many cycles of the CV and LSV. The results revealed that Al, Ce, Ti, and Ba concentration (< 0.1, < 0.1, 0.07, and 0.067 µg L^−1^, respectively) was negligible. The obtained results not only illustrate the stability of our electrocatalyst but also highlight the heterogeneous reactions during the electrochemical processes.

## Conclusion

In summary, based on the results of our study, we have demonstrated the successful doping of Al and Ce into the structure of BaTiO_3_, which has resulted in significant improvements in the electrocatalytic activity of the material. Accordingly, the electrocatalysts were synthesized via a two-step hydrothermal method. The XRD, FT-IR, Raman, XPS, SEM, TEM and HR-TEM, TEM- mapping and EDX were utilized to characterize the as-prepared nanofiber materials. The analysis demonstrated the successful synthesis and appropriate introduction of the Al and Ce dopant into the BaTiO_3_ lattice. The unique combination of these dopants has resulted in a novel electrocatalyst that exhibits excellent performance in various electrochemical processes such as OER, HER, and overall water splitting. By co-doping BaTiO_3_ with Al and Ce, the overpotential values for both OER and HER processes were observed to be highly active, with values of − 894 and − 570 mV vs. RHE respectively, indicating the excellent performance of the Al-Ce co-doped BaTiO_3_ electrode. The high performance of the Al-Ce co-doped BaTiO_3_ electrode was demonstrated by its low onset potentials in both the oxygen evolution reaction (OER) (11 mV vs. RHE) and hydrogen evolution reaction (HER) (− 174 mV vs. RHE). Furthermore, the electrode displayed remarkable efficiency in overall water splitting, with an overpotential of only 0.820 mV at a current density of 10 mA cm^−2^. These results indicate the excellent potential of this electrode for practical applications in various electrochemical devices. In addition, the Al-Ce co-doped BaTiO_3_ electrode exhibited high capacitance as a pseudo-capacitor, with a value of 224.18 Fg^−1^ at a scan rate of 10 mVs^−1^, and excellent durability over 100 cycles at 100 mVs^−1^. Additionally, the durability of the electrode was confirmed by the SEM image after undergoing 600 cycles of LSV at a scan rate of 10 mVs^−1^. In a conclusion, our results demonstrate the effectiveness of this novel electrocatalyst and its potential as a low-cost alternative to noble metal-based electrocatalysts, making it an essential contribution to the field of sustainable energy.

## Methods

### Applied chemical compounds and instruments

TiO_2_ (99.9%, Sigma Aldrich, USA), NaOH solution (10 mM, ≥ 98%, Sigma Aldrich, USA), HCl (0.2 M, 37%, Merck, Germany), Ba (OH)_2_.8H_2_O (> 98%, Sigma Aldrich, USA), Al (OH)_3_ (98%, Sigma Aldrich, USA), Ce (NO_3_)_2_ (99.99%, Sigma Aldrich, USA), and Nafion (5%, Sigma Aldrich, USA) were used for the synthesis and application of Al-Ce co-doped BaTiO_3_. All chemicals were analytically pure and were used without further purification.

The X-ray diffraction (XRD) patterns were determined by Bruker D8 Advance X-ray diffractometer built in Germany with Cu Kα radiation. The FT-IR spectrum was measured using a Bruker Tensor-27 manufactured in Germany. Raman spectrum were collected using a Uninanotech model Unidron-A made in Korea. X-ray photoelectron spectroscopy (XPS) measurements were performed using a Kratos AXIS UltraDLD Thermo scientific photoelectron spectrometer produced in the UK. Scanning electron microscopy (SEM) images were acquired using a Tescan MIRA 3 microscope created in the Czech Republic. High-resolution transmission electron microscopy (HRTEM) images were obtained using a JEM-2100, Jeol microscope manufactured in Japan, equipped with an energy-dispersive X-ray (EDX) spectrometer. The cyclic voltammetry (CV) and linear seep potentiostat LSV experiments were conducted using an AUTO LAB PGSSTAT100 made in the Netherlands. Finally, the impedance and galvanostatic charge–discharge (GCD) experiments were performed using an Origa OGF500 system fabricated in France.

**Figure 5:**
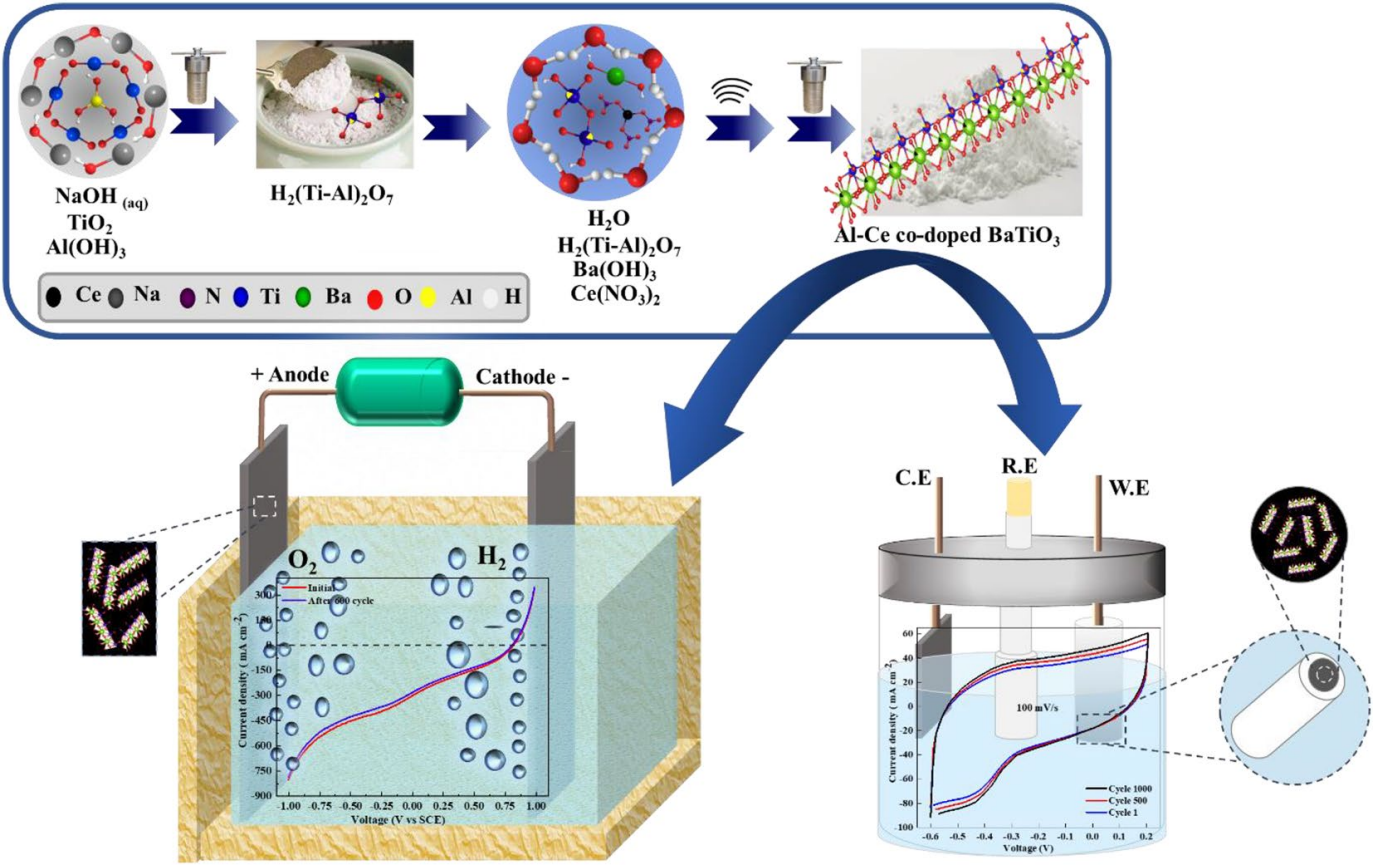
The schematic diagram of the synthesis and application of the Al-Ce co-doped BaTiO_3._

### Synthesis of pure and Al-Ce co-doped BaTiO_3_

BaTiO_3_ nanofibers were synthesized utilizing a two-step hydrothermal method described in the previously published paper^42^. First, 1500 mg of TiO_2_ was dissolved in a NaOH solution (10 M) and agitated for 2 h to produce the white homogeneous suspension. Later, the above solution was moved into the 100 mL Ce (NO_3_)_3_ Teflon-lined hydrothermal synthesis autoclave and heated for 24 h at 200 °C. Afterward, the obtained white spongy texture H_2_Ti_3_O_7_ NFs precipitate^42^ was filtered and washed with DI water and soaked in HCl (0.2 M) for three hours. Next, the aforementioned precipitate was centrifuged with DI water three times and dried at 70 °C for 12 h. The collected sample was then used for the synthesis of BaTiO_3_ NFs. Hence, 150 mg of as-prepared H_2_Ti_3_O_7_ NFs and 70 mL of Ba (OH)_2_. 8H_2_O solution was sonicated for 20 min. Accordingly, the resulting solution was transferred to the 100 mL Teflon-lined hydrothermal synthesis autoclave and heated for 24 h at 200 °C. Then, the obtained white precipitation was soaked in the solution of HCl (0.2 M) for 3 h^30^. Finally, the white BaTiO_3_ precipitation was washed with DI water, centrifuged three times, and dried at 70 °C for 12 h.

To synthesize the Al-Ce doped BaTiO_3_, the same procedure as that of BaTiO_3_ was followed with some modifications. Al and Ce were added in different steps based on their oxidation states and ionic radius^79^, in order to substitute for Ti and Ba, respectively^80^. In the first step of the hydrothermal synthesis method, 42 mg Al (OH)_3_ was added to the NaOH solution, while in the second step, 9.3 mg of Ce (NO_3_)_2_ was added to the solution of Ba (OH)_2._ 8H_2_O to prepare the Al_0.03_-Ce_0.03_ co-doped Ba_0.97_ Ti_0.97_O_3_. Furthermore, the same synthesis method was used for the preparation of Al-doped or Ce-doped BaTiO_3_.

### Electrochemical experiments

To prepare the working electrode, 10 mg of BaTiO_3_ powder was dispersed in 1.5 mL of ethanol for 10 min. Next, 5 µL of diluted Nafion (2.0% wt) was added to the solution and sonicated for 30 min to achieve a well dispersed suspension. A homogeneous suspension of 5 µL was drop-casted onto a pristine graphite with a surface area of 0.096 cm^2^ and dried, resulting in a BaTiO_3_ electrocatalyst loading of 0.552 mg cm^−2^. The same procedure was repeated for the preparing Al-doped BaTiO_3,_ Ce-doped BaTiO_3,_ and Al-Ce co-doped BaTiO_3_ electrodes. After each test, the electrodes were polished with Al_2_O_3_ powder, sonicated for 30 min in H_2_O/Methanol 50:5 solution, and cleaned with DI water to prepare for the next tests. Moreover, the Eq. (10) applied for converting the SCE unit to RHE:

10 $${\text{E }}\left( {\text{vs RHE}} \right) \, = {\text{ E }}\left( {\text{vs SCE}} \right) \, + \, 0.{244 } + \, 0.0{\text{59pH}}$$

In the above-mentioned Eq, the pH related to the pH of the electrolyte. In this study, we used the 1 M KOH solution. Therefore, the pH was 14. Moreover, the onset potential means the potential where the current starts increasing from zero value. On the other hand, when we modify the diagrams according to the RHE, the origin of the diagram alters to 1.07 based on Eq. (10)^81^. Thus, we calculated the distances between the origin of the diagram and the current starting increasing (∆E) in both OER and HER reactions as the onset potential. Further, Overpotential is typically assessed at a given current density (most commonly at 10 and − 10 mA cm^−2^)^82^.

To facilitate the overall water splitting process, two 30 × 30 × 0.5 mm graphite pieces were coated with 450 µL of the Al-Ce co-doped BaTiO_3_ ink on each side, to serve as electrodes. The coated graphite pieces were dried at 80 °C to ensure the electrocatalyst was evenly distributed on the electrode surface. The schematic diagram of the synthesis and application of the Al-Ce co-doped BaTiO_3_ is shown in Fig. 5._._

## Supplementary Information

Supplementary Information.

## Data Availability

The data that support the findings of this study are available from the corresponding author on reasonable request.
