# Supplementary material for: Al-Ce co-doped BaTiO3 nanofibers as a high-performance bifunctional electrochemical supercapacitor and water-splitting electrocatalyst
Source: Sci Rep. 2024 Apr 29;14:9833. doi: 10.1038/s41598-024-54561-4 (PMC11538466; doi:10.1038/s41598-024-54561-4)
Supplement: Supplementary file 1 — Supplementary Information. [file 41598_2024_54561_MOESM1_ESM.docx]

**Supplementary materials**

**Al-Ce co-doped BaTiO_3_ nanofibers as a high-performance bifunctional electrochemical supercapacitor and water-splitting electrocatalyst**

**Table. S1. Elemental content of** BaTiO_3_ and Al-Ce co-doped BaTiO_3_ by XPS analysis.

| Electrocatalyst | Elements | Atomic % |
| --- | --- | --- |
| BaTiO_3_ | **Ba 3d**  **Ti 2p**  **O 1s**  **C 1s** | **22.95**  **21.41**  **52.44**  **3.20** |
| Al-Ce co-doped BaTiO_3_ | **Ba 3d**  **Ti 2p**  **O 1s**  **Al 2p**  **Ce 3d**  **C 1s** | **22.23**  **20.81**  **53.44**  **0.60**  **0.72**  **2.20** |


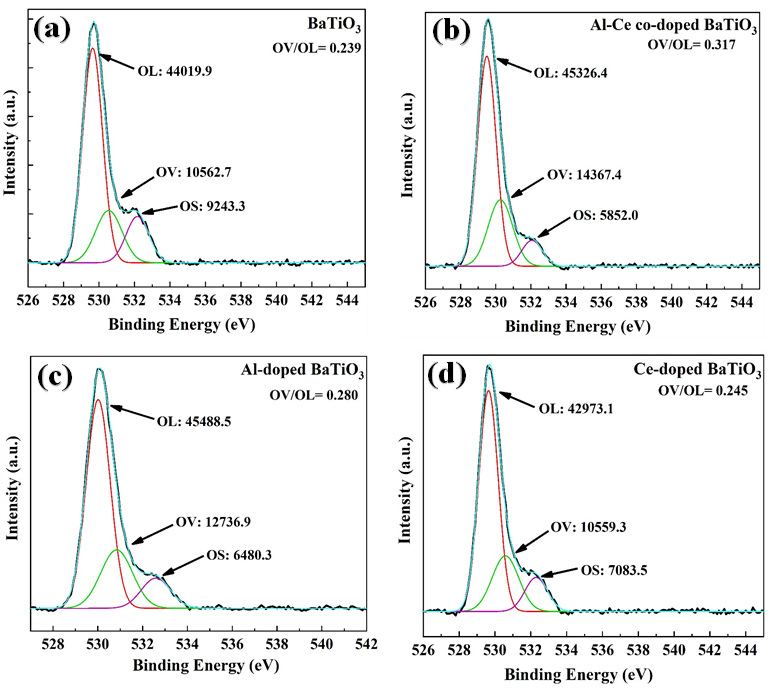


**Fig.S1.** Structural characterization of the so-synthesized materials. High-resolution XPS spectra of O 1s of bare BaTiO_3_ (a), Al- doped BaTiO_3_ (b), Ce- doped BaTiO_3_ (c)_,_ and Al-Ce co-doped BaTiO_3_ (d).

**Fig.S2.** (a-b) The SEM and (c-d) TEM of Al-doped BaTiO_3_ and Ce-doped BaTiO_3_ respectively. (e) EDX plot and (f-k) TEM-mapping of Al-Ce co-doped BaTiO_3._
